# Dietary Cholesterol Intake and Risk of Lung Cancer: A Meta-Analysis

**DOI:** 10.3390/nu10020185

**Published:** 2018-02-08

**Authors:** Xiaojing Lin, Lingli Liu, Youyun Fu, Jing Gao, Yunyun He, Yang Wu, Xuemei Lian

**Affiliations:** 1 School of Public Health and Management, Research Center for Medicine and Social Development, Innovation Center for Social Risk Governance in Health, Chongqing Medical University, Chongqing 400016, China; 2015111087@stu.cqmu.edu.cn (X.L.); 2015111098@stu.cqmu.edu.cn (L.L.); 2016111039@stu.cqmu.edu.cn (Y.F.); 2016111060@stu.cqmu.edu.cn (J.G.); 2015111099@stu.cqmu.edu.cn (Y.H.); 2 Center for Lipid Research, Key Laboratory of Molecular Biology for Infectious Diseases (Ministry of Education), Chongqing 400016, China; 3 College of Stomatology, Chongqing Medical University, Chongqing 401147, China; yangwu@hospital.cqmu.edu.cn; 4 Chongqing key Laboratory of Oral Diseases and Biomedical Sciences, Chongqing 401147, China

**Keywords:** dietary cholesterol, lung cancer, cholesterol metabolism, case-control study, cohort study, meta-analysis

## Abstract

Multiple epidemiologic studies have evaluated the relationship between dietary cholesterol and lung cancer risk, but the association is controversial and inconclusive. A meta-analysis of case-control studies and cohort studies was conducted to evaluate the relationship between dietary cholesterol intake and lung cancer risk in this study. A relevant literature search up to October 2017 was performed in Web of Science, PubMed, China National Knowledge Infrastructure, Sinomed, and VIP Journal Integration Platform. Ten case-control studies and six cohort studies were included in the meta-analysis, and the risk estimates were pooled using either fixed or random effects models. The case-control studies with a total of 6894 lung cancer cases and 29,736 controls showed that dietary cholesterol intake was positively associated with lung cancer risk (Odds Ratio = 1.70, 95% Confidence Interval: 1.43–2.03). However, there was no evidence of an association between dietary cholesterol intake and risk of lung cancer among the 241,920 participants and 1769 lung cancer cases in the cohort studies (Relative Risk = 1.08, 95% Confidence Interval: 0.94–1.25). Due to inconsistent results from case-control and cohort studies, it is difficult to draw any conclusion regarding the effects of dietary cholesterol intake on lung cancer risk. Carefully designed and well-conducted cohort studies are needed to identify the association between dietary cholesterol and lung cancer risk.

## 1. Introduction

Lung cancer is one of the most common cancers. Cancer epidemiological data showed that, in 2012, there were about 1.8 million new lung cancer cases and 1.6 million cases of death, respectively, accounting for about 13% of the total number of cancer diagnosis and 20% of the total number of cancer deaths [1]. However, early detection of lung cancer is very difficult, and prevention has become the main method to reduce the incidence of lung cancer. Smoking is currently the most important risk factor for lung cancer, but there are still about 25% of lung cancer patients that are non-smokers [2]. Dietary adjustment represents another important factor in the prevention of cancer. Baena et al. reviewed the published epidemiological studies and found a direct relationship between dietary factors and cancer development risks, accounting for up to 35% of the risk factors [3].

The dietary factors and lung cancer risk studies that have found arsenic in drinking water and (in smokers only) pharmacological doses of beta-carotene to be causes of lung cancer are convincing. Fruits, as well as foods containing carotenoids, probably protect against lung cancer [4,5]. Cholesterol is a necessary compound for maintaining cellular homeostasis. Recently, emerging in vivo animal studies have connected intracellular cholesterol metabolism disturbance with lung diseases, including lung cancer [6]. Cholesterol levels tend to be high in intracellular cancer cells and cholesterol is needed for cancer progression [7]. Since a diet high in cholesterol might be indicative of a lifestyle prone to health-related problems such as cardiovascular diseases, cancer, etc., the association between dietary cholesterol and cancer risk has recently received considerable attention [8,9]. However, the relationship between dietary cholesterol intake and lung cancer risk is currently inconclusive. Most case-control studies suggested that high cholesterol intake may increase lung cancer risk, but cohort studies showed different results. The objective of this meta-analysis was to systematically analyze the relationship between dietary cholesterol intake and the risk of lung cancer.

## 2. Materials and Methods

### 2.1. Literature Search

A systematic literature search up to October of 2017 was performed in Pubmed, Web of Science, China National Knowledge Infrastructure (CNKI), Sinomed, and VIP Journal Integration (VIP) to identify relevant studies. Search terms included: “cholesterol” and “lung cancer” or “lung carcinoma” or “cancer of lung” or “lung neoplasm”. Moreover, we reviewed reference lists of retrieved articles to identify any potentially relevant studies.

### 2.2. Study Selection

Eligible trials were selected by two reviewers independently. Disagreement between the two reviewers was resolved by discussing with a third reviewer. Inclusion criteria were: (1) exposure factor was dietary cholesterol, and language was limited to Chinese and English; (2) studies designed as a case-control study or cohort study; (3) the outcome of interest was risk of lung cancer; and (4) relative risk (RR), odds risk (OR), or Hazard Ratio (HR) estimates with 95% confidence intervals.

### 2.3. Data Extraction

A purpose-designed form was used by two independent reviewers to collect the following data: first author, publication date, country, sample size, sex of subjects, dietary cholesterol intake level, other lung cancer risk factors adjusted, and effect estimates with corresponding 95% CIs for the highest versus the lowest categories of dietary cholesterol intake levels.

### 2.4. Quality Assessment

We chose to use the nine-star Newcastle-Ottawa Scale (NOS) to assess the methodological quality of case–control and cohort studies [10]. The NOS scale consists of three dimensions: selection (four stars), comparability (two stars), and exposure/outcome (three stars).

### 2.5. Statistical Analysis

On the basis of study design, two separate meta-analyses were conducted: one for case-control studies and the other for cohort studies. For the quantification of the ORs (or HRs) in our meta-analyses, we extracted ORs (or HRs or RRs) comparing the extreme categories of dietary cholesterol intake (highest compared with lowest) as defined within each study. If multiple ORs (or HRs) were analyzed in one study, we extracted risk estimates from the greatest degree of control for potential confounding factors. Stata version 12.0 (StataCorp, College Station, TX, USA) was used for statistical analysis. Heterogeneity was assessed using the method of Cochran Q and *I*^2^ statistics. For the Q statistic, statistically significant for heterogeneity was set as *p* value < 0.1. The *I*^2^ test was used to provide further evidence of heterogeneity. A random-effects model was adopted for meta-analysis if heterogeneity was detected; otherwise, a fixed-effects model was employed. Then the Egger’s test and Begg’s test were used to analyze the publication bias. Finally, a sensitivity analysis was performed.

## 3. Results

### 3.1. Literature Search and Study Characteristics

**Figure 1:**
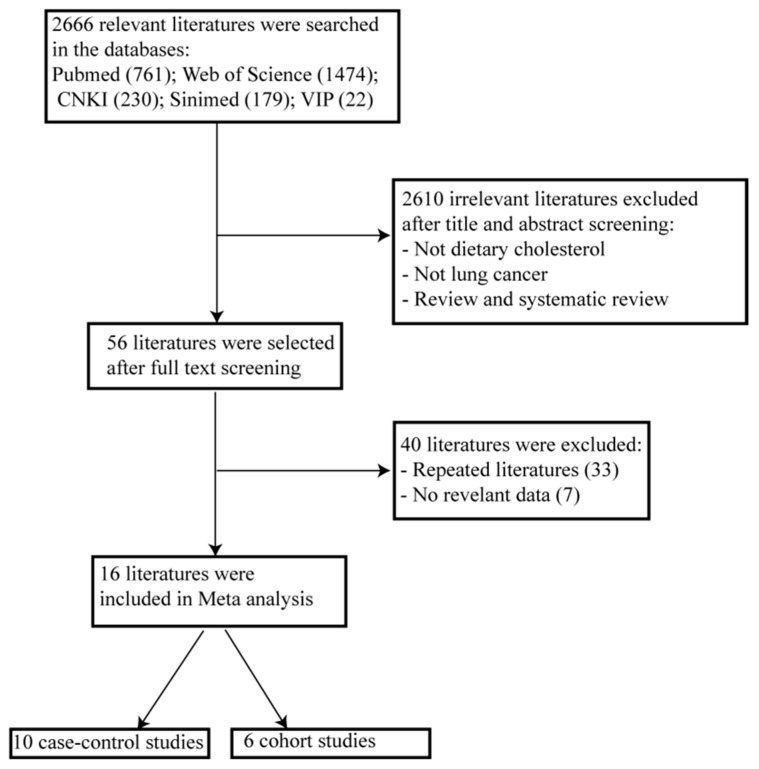
Flow diagram of the literature selection.

Figure 1 shows the flow diagram of study inclusion. A total of 2666 relevant studies were identified during the initial search. We identified 56 studies on the basis of the title and abstract. After detailed evaluation, 40 studies were excluded for reasons described in Figure 1. Finally, the remaining 16 studies were included in the meta-analysis, with 10 case-control studies and six cohort studies. Other details of the baseline data are shown in Table 1 and Table 2. A total of 6894 lung cancer cases and 29,736 controls were included in the 10 case-control studies, and 241,920 participants, including 1769 lung cancer cases, were included in the six cohort studies. The Newcastle-Ottawa Scale scores for the included studies ranged from 6 to 9, and all studies were deemed to be of high quality (≥6).

### 3.2. Data Analysis

#### 3.2.1. Dietary Cholesterol and Lung Cancer Risk

**Figure 2:**
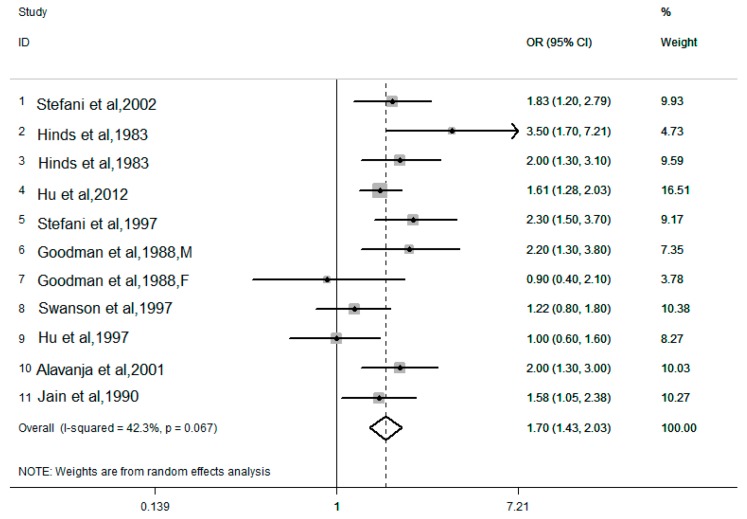
Forest plot of the highest compared with the lowest categories of intake of the dietary cholesterol and lung cancer risk in 10 case-control studies.

**Figure 3:**
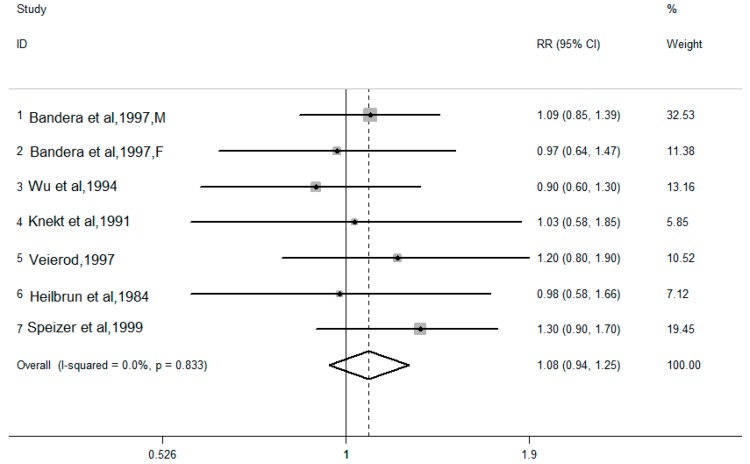
Forest plot of the highest compared with the lowest categories of intake of dietary cholesterol and lung cancer risk in six cohort studies.

The pooled results combined for the highest versus lowest dietary cholesterol intake levels are shown in Figure 2 and Figure 3. There are 10 case-control studies, with a total of 6894 lung cancer cases and 29,736 controls. There was moderate heterogeneity in the results of the association between dietary cholesterol (*I*^2^ = 42.3%, *P* for heterogeneity = 0.067) and lung cancer risk. Using a random effects model, the pooled OR for the highest and lowest levels of dietary cholesterol was 1.70 (95% CI: 1.43–2.03). Six cohort studies provided the dietary cholesterol intake levels and lung cancer risk, with a total of 241,920 participants and 1769 lung cancer cases. There was no heterogeneity in the results of the association between dietary cholesterol (*I*^2^ = 0.0%, *P* for heterogeneity = 0.833) and lung cancer risk. Using a fixed effects model, the pooled RR for the highest and lowest levels of dietary cholesterol was 1.08 (95% CI: 0.94–1.25).

#### 3.2.2. Dietary Total Fat and Lung Cancer Risk

**Figure 4:**
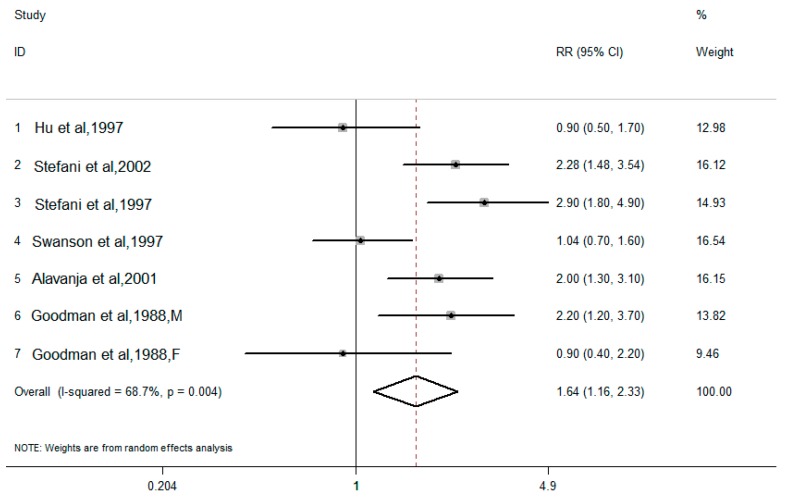
Forest plot of the highest compared with the lowest categories of intake of dietary total fat and lung cancer risk in six case-control studies.

Among the 10 case-control studies, there were six case-control studies that also analyzed dietary total fat and lung cancer risk. The pooled results combined for the highest versus the lowest dietary total fat intake levels, and are shown in Figure 4. There was moderate heterogeneity in the results of the association between dietary total fat (*I*^2^ = 68.7%, *P* for heterogeneity = 0.004) and lung cancer risk. Using a random effects model, the pooled OR for the highest and lowest levels of dietary total fat was 1.64 (95% CI: 1.16–2.33).

### 3.3. Publication Bias

The Egger’s test and Begg’s test were used to analyze the publication bias. There was no evidence of publication bias observed. Case-control studies: Egger’s test, *p* = 0.737, Begg’s test, *p* = 0.213; cohort studies: Egger’s test, *p* = 0.459, Begg’s test, *p* = 1.000.

### 3.4. Sensitivity Analysis

To test the stability and credibility of the results from the dietary cholesterol and lung cancer risk meta-analysis, we recalculated the pooled effect measure after systematically removing each study and found no significant change in the results, indicating that our findings are stable and credible.

## 4. Discussion

The objective of this study was to use meta-analysis to evaluate the correlation between dietary cholesterol and lung cancer risk. An analysis of 6894 lung cancer cases and 29,736 controls in 10 case-control studies showed that the dietary cholesterol intake levels (OR = 1.70, 95% CI: 1.43–2.03) were positively associated with lung cancer risk. An analysis of 1769 lung cancer cases among 241,920 participants in six cohort studies showed that there was no association between dietary cholesterol intake levels and lung cancer risk (RR = 1.08, 95% CI: 0.94–1.25); this is the same as the Smith-Warner’s pooled analysis of cohort studies [27].

However, we found that the results of case-control studies and cohort studies were inconsistent in our meta-analysis. Although a positive correlation between dietary cholesterol intake and lung cancer risk was found in 10 case-control studies, the result needs to be carefully interpreted. Dietary cholesterol intake is often accompanied by dietary fat intake. Yang et al. [28] conducted a pooled analysis of dietary fat intake and lung cancer risk and found that total fat intake was associated with an increased risk of lung cancer. To further explore the results of our case-control studies, six studies including dietary total fat were specifically analyzed, and we found that dietary total fat intake levels were positively associated with lung cancer risk (OR = 1.64, 95% CI: 1.16–2.33). Therefore, a strong positive correlation between dietary fat and lung cancer risk might mask the relationship between dietary cholesterol and lung cancer risk. Moreover, case-control studies are prone to bias, such as recall bias, selectivity bias, and confounding factors that are more difficult to control. It is difficult to determine whether or not patients modify their diet or their dietary recall while under treatment for lung cancer. Then, selected cases or control groups could not represent the population, and the conclusion cannot be deduced to represent the whole population. Most importantly is that the case-control study can only preliminarily test the possible association, but not a causal relationship between factors and the disease [29]. With higher strength of evidence, prospective cohort studies could analyze the role of the dietary factors more fully and directly, and may give a hint to a causal link [30]. From a systematic meta-analysis of six cohort studies, we found no statistically significant association between dietary cholesterol and lung cancer risk. The result is quite reasonable if we realize that there are conception differences among intrapulmonary cholesterol, serum cholesterol, and dietary cholesterol.

Cholesterol is an essential compound for many physiological processes in the body, and an important lipid for maintaining cellular homeostasis. Even though conflicting epidemiologic evidence leads to uncertainty regarding a role for cholesterol in cancer development, it is consistent that cholesterol levels tend to be high in cancer cells and intracellular cholesterol is needed for cancer progression [7]. Recently, emerging in vivo animal studies have connected intracellular cholesterol metabolism disturbance with lung diseases, including lung cancer [6]. Liver X receptors (LXRs) are best known as cellular cholesterol sensors which physiologically regulate intracellular cholesterol homeostasis. The ablation of liver X receptors α and β was reported to cause peripheral squamous cell lung cancer spontaneously in mice [31]. Our previous study found that an atherogenic diet high in cholesterol content (HCD) might play a protection role in urethane-induced lung carcinogenesis in C57BL/6J mice. Even though hypercholesterolemia presented in HCD-fed mice, the intrapulmonary cholesterol levels decreased significantly in urethane-treated HCD-fed mice compared to control diet-fed mice [32]. LXR activation in the urethane-treated HCD-fed mice mediated cholesterol efflux from the lung and played an important role in maintaining pulmonary cholesterol homeostasis. In summary, intrapulmonary cholesterol levels are most critical in lung cancer development.

Serum total cholesterol (TC) was commonly used as a biomarker of cholesterol metabolic status in the body, especially for the risk of cardiovascular diseases. For the relationship between the serum TC level and lung cancer risk, an inverse relationship was observed in multiple case-control and cohort studies. If we excluded the impact of pre-diagnostic cancer progress on serum TC levels, a significantly inverse association between total cholesterol and lung cancer risk was observed from our recently-published meta-analysis (RR = 0.89, 95% CI: 0.83–0.94) [33]. Therefore, it is highly possible that an etiological relationship exists between cholesterol metabolism and lung cancer development, and there might be specific metabolic pathways of cholesterol in the lung [32]. The main sources of cholesterol in the body are from in vivo synthesis and food intake; even though meta-analysis found that each additional 100 mg of dietary cholesterol in the diet resulted in a change of 2.2 mg/dL in serum total cholesterol levels [34], the effect of dietary cholesterol intakes on blood cholesterol levels presented great individual variation, and is related to dietary compositions. It is even harder to decide the intrapulmonary cholesterol level according to dietary intake. Therefore, it is quite reasonable that there is no statistically significant association between dietary cholesterol intake and lung cancer risk observed in this meta-analysis of cohort studies. Likewise, although serum cholesterol level is highly correlated to cardiovascular disease (CVD) risk, multiple epidemiological studies and clinical interventions have shown that a lack of correlation between cholesterol intake and CVD risk or death were observed [35].

We acknowledge that the major limitations of this study may include not accessing individual participant data and inter-study variability in cut-off values of cholesterol. And cohort studies used just one baseline measurement of cholesterol intake across years of follow up (regression dilution bias) could possibly account for some disparity between case-control and cohort studies.

## 5. Conclusions

Due to inconsistent results from cohort and case-control studies, it is difficult to draw any conclusions regarding the effects of dietary cholesterol intake on lung cancer risk. With concerns about the interaction between cholesterol metabolism-related genetic variants and dietary cholesterol intakes, carefully designed and well-conducted cohort studies are needed to elucidate the relationship between dietary cholesterol and lung cancer risk.

## Figures and Tables

**Table 1:** Case-control studies included in the meta-analysis.

Author	Year	Location	Cases	Controls	Gender	Adjusted OR (95% CI)	Dietary Cholesterol (Tool)	Quality	Adjustments
Hinds et al. [11]	1983	USA	188	294	Male Female	3.50 (1.70–7.21)	>3500 mg/week vs. <999 mg/week (FFQ)	6	Ethnicity, age, pack-years of cigarettes smoked, and occupational exposure to carcinogens
Hinds et al. [12]	1983	USA	364	627	Male Female	2.0 (1.3–3.1)	>2071 mg/week vs. <750 mg/week (FFQ)	7	Vitamin A intake, sex, age, pack-years of smoking, ethnicity, occupational status
Goodman et al. [13]	1988	USA	326	865	Male	2.2 (1.3–3.8)	Q4 vs. Q1 (FFQ)	7	Age, ethnicity, and pack-years of cigarette smoking
					Female	0.9 (0.4–2.1)			
Jain et al. [14]	1990	Canada	839	772	Male Female	1.58 (1.05–2.38)	>468 mg/day vs. <234 mg/day (FFQ)	7	Age and cumulative cigarette smoking
Stefani et al. [15]	1997	Uruguay	426	419	Male	2.3 (1.5–3.7)	>610.7 mg/day vs. ≤354.6 mg/day (FFQ)	7	Age, residence, urban/rural status, tobacco smoking, total energy intake, a term for all vegetables and fruits and α-carotene intake
Swanson et al. [16]	1997	USA	624	587	Female	1.22 (0.8–1.8)	>177 mg/1000 Kcal vs. <102 mg/1000 Kcal (FFQ)	6	Age and total calories, education, pack-years of smoking, body mass index, consumption of vegetables and fruit
Hu et al. [17]	1997	China	227	227	Male Female	1.0 (0.6–1.6)	>108.22 mg/day vs. <19.02 mg/day ( FFQ)	8	Cigarettes per day, duration, and family income
Alavanja et al. [18]	2001	USA	360	574	Female	2.0 (1.3–3.0)	Q5 vs. Q1 (FFQ)	7	Age and nutrient density calories
Stefani et al. [19]	2002	Uruguay	200	600	Male	1.83 (1.20–2.79)	T3 vs. T1 (FFQ)	7	Age, residence, urban/rural status, education, body mass index, smoking status, smoking duration, and total energy intake
Hu et al. [20]	2012	Canada	3341	24,771	Male Female	1.61 (1.28–2.03)	≥1880.266 mg/week vs. ≤966.261 mg/week (FFQ)	7	Sex, age group, province, education, body mass index, alcohol drinking, pack-years of smoking, total of vegetable and fruit intake, saturated fat and total energy intake

OR, odds ratio; CI: confidence interval; FFQ, food frequency questionnaire. Q1, quartile 1; Q4, quartile 4; T1, tertile 1; T3, tertile 3.

**Table 2:** Cohort studies included in the meta-analysis.

Author	Year	Location	Follow-Up	No. of Cases/Participants	Gender	Adjusted RR (95% CI)	Dietary Cholesterol (Tool)	Quality	Adjustments
Heilbrun et al. [21]	1984	USA	1968–1983	113/7539	Male	0.98 (0.58–1.66)	>750 mg/day vs. <299 mg/day (24h recall )	9	Age and pack-years of smoking
Knekt et al. [22]	1991	Finland	1967–1986	117/4538	Male	1.03 (0.58–1.85)	>609 mg/day vs. <441 mg/day (FFQ)	8	Age, smoking, and energy intake
Wu et al. [23]	1994	USA	1985–1991	272/41,837	Female	0.9 (0.6–1.3)	>365.5 mg/day vs. <200.5 mg/day (FFQ)	8	Age, smoking status, pack-years of cigarettes, occupation, physical activity, and total energy intake
Bandera et al. [24]	1997	USA	1980–1987	525/48,000	Male	1.09 (0.85–1.39)	T3 vs. T1 (FFQ)	9	Age, education, cigarettes/day, years smoking, and total energy intake (except calories)
					Female	0.97 (0.64–1.47)			
Veierød et al. [25]	1997	Norway	1980–1991	149/50,712	Male Female	1.2 (0.8–1.9)	≥240.6 mg/day vs. ≤154.9 mg/day (FFQ)	9	Smoking status, gender, age at inclusion and attained age
Speizer et al. [26]	1999	USA	1980–1992	593/89,294	Female	1.3 (0.9–1.7)	Q5 vs. Q1 (FFQ)	8	Age, total energy intake, smoking, and age of starting to smoke

RR, relative risk; CI, confidence interval; FFQ, food frequency questionnaire; T1, tertile 1; T3, tertile 3; Q1, quintile 1; Q5, quintile 5.
